# Progressive mitochondrial protein lysine acetylation and heart failure in a model of Friedreich’s ataxia cardiomyopathy

**DOI:** 10.1371/journal.pone.0178354

**Published:** 2017-05-25

**Authors:** Amanda R. Stram, Gregory R. Wagner, Brian D. Fogler, P. Melanie Pride, Matthew D. Hirschey, R. Mark Payne

**Affiliations:** 1 Department of Surgery, Indiana University School of Medicine, Indianapolis, Indiana, United States of America; 2 Department of Cellular & Integrative Physiology, Indiana University School of Medicine, Indianapolis, Indiana, United States of America; 3 Herman B. Wells Center for Pediatric Research, Indiana University School of Medicine, Indianapolis, Indiana, United States of America; 4 Division of Endocrinology, Metabolism, and Nutrition, Duke University, Durham, North Carolina, United States of America; 5 Department of Pediatrics, Indiana University School of Medicine, Indianapolis, Indiana, United States of America; Scuola Superiore Sant'Anna, ITALY

## Abstract

**Introduction:**

The childhood heart disease of Friedreich’s Ataxia (FRDA) is characterized by hypertrophy and failure. It is caused by loss of frataxin (FXN), a mitochondrial protein involved in energy homeostasis. FRDA model hearts have increased mitochondrial protein acetylation and impaired sirtuin 3 (SIRT3) deacetylase activity. Protein acetylation is an important regulator of cardiac metabolism and loss of SIRT3 increases susceptibility of the heart to stress-induced cardiac hypertrophy and ischemic injury. The underlying pathophysiology of heart failure in FRDA is unclear. The purpose of this study was to examine in detail the physiologic and acetylation changes of the heart that occur over time in a model of FRDA heart failure. We predicted that increased mitochondrial protein acetylation would be associated with a decrease in heart function in a model of FRDA.

**Methods:**

A conditional mouse model of FRDA cardiomyopathy with ablation of FXN (FXN KO) in the heart was compared to healthy controls at postnatal days 30, 45 and 65. We evaluated hearts using echocardiography, cardiac catheterization, histology, protein acetylation and expression.

**Results:**

Acetylation was temporally progressive and paralleled evolution of heart failure in the FXN KO model. Increased acetylation preceded detectable abnormalities in cardiac function and progressed rapidly with age in the FXN KO mouse. Acetylation was also associated with cardiac fibrosis, mitochondrial damage, impaired fat metabolism, and diastolic and systolic dysfunction leading to heart failure. There was a strong inverse correlation between level of protein acetylation and heart function.

**Conclusion:**

These results demonstrate a close relationship between mitochondrial protein acetylation, physiologic dysfunction and metabolic disruption in FRDA hypertrophic cardiomyopathy and suggest that abnormal acetylation contributes to the pathophysiology of heart disease in FRDA. Mitochondrial protein acetylation may represent a therapeutic target for early intervention.

## Introduction

Heart disease and heart failure exert a significant morbidity and mortality burden worldwide. Abnormal metabolism and metabolic remodeling are common pathologic features in heart disease, such as in obesity and diabetes-related cardiomyopathy [1, 2], ischemia [3], and heart failure [4, 5]. Mitochondrial dysfunction and heart disease are closely linked [6], and because mitochondria are central to regulating cellular energy and fulfilling the high demands of cardiac metabolism, it is expected that mitochondrial dysfunction plays a key pathologic role in abnormal cardiac metabolism [7].

Mitochondrial protein acetylation is an important post-translational regulatory mechanism of heart metabolism that has emerged in recent years. Acetylation in mitochondria is controlled by the NAD^+^-dependent deacetylase, sirtuin 3 (SIRT3). SIRT3 responds to cellular energy status and utilizes an “acetylation switch” to modify protein function [8] in order to respond quickly to metabolic cues and thus, operates a chief mechanism for maintaining normal mitochondrial function and metabolism [9]. SIRT3 targets enzymes that are typically activated in response to removal of acetyl groups, and which are important to energy generation and utilization, such as very-long-chain, long-chain, and medium-chain acyl-CoA dehydrogenases (VLCAD, LCAD and MCAD) [10, 11], pyruvate dehydrogenase (PDH) [12], acetyl CoA synthetase 2 (AceCS2) [13], and electron transport chain (ETC) complexes I-III [14–16]. Additionally, SIRT3 appears to deacetylate other important proteins, such as those that provide protection from oxidative damage, e.g., superoxide dismutase 2 (SOD2) [17]. It is worth noting here that enzyme response to acetylation state is an area of continuing investigation. For instance, others have reported an increase in activity in LCAD in response to acetylation, specifically in obesity-related heart disease [18].

Beneficial effects of SIRT3 activity in the heart are well documented. For example, SIRT3 has been shown to protect against oxidative stress, attenuate fatty acid accumulation in the heart [19], and prevent development of stress-induced cardiac hypertrophy [20]. Mice lacking SIRT3 have mitochondrial dysfunction eventually resulting in myocardial energy loss, develop hypertrophy and fibrosis in response to mechanical stress [21], and are more susceptible to the detrimental effects of ischemia/reperfusion injury [22].

Acetylation is most exciting in the context of heart disease in that it has potential as a therapeutically modifiable target. For example, recent work has shown that exogenous treatment with NAD^+^ precursors can increase NAD^+^ levels in mitochondria, activate sirtuins to reduce protein acetylation, and improve cardiac outcome [23, 24].

The heart disease of Friedreich’s Ataxia (FRDA) results from inherited deficiency of frataxin (FXN), a mitochondrial protein important in energy homeostasis. FXN is a highly conserved mitochondrial matrix protein that functions in iron-sulfur cluster assembly, which is integral to mitochondrial metabolic machinery [25]. Reduced expression of FXN in FRDA results in impaired energy generation, a decreased NAD^+^/NADH ratio, and increased oxidative stress [26–28]. In addition to ataxia, patients often develop hypertrophic cardiomyopathy and heart failure. The vast majority of patients who go on to develop cardiomyopathy are asymptomatic until late stages of disease. The most frequent cause of mortality in FRDA arises from cardiac etiology, most commonly due to congestive heart failure [29]. There is no known cure.

Mice with conditional loss of FXN in the heart develop cardiac hypertrophy as early as 5 weeks of age, followed by transition to dilated cardiomyopathy and heart failure by approximately 8 weeks of age [30]. Certain biochemical and structural changes occur in the FRDA mouse model heart as early as 4 weeks of age, including reduced activity of important metabolic enzymes (such as those of the ETC complexes and aconitase) and mitochondrial ultrastructure abnormalities [30]. The importance of these findings is that underlying changes in the FRDA heart originate prior to overt abnormalities in cardiac function and thus, may present a window of opportunity for intervention before irreversible changes occur.

We previously reported that mitochondrial proteins in FRDA mouse model hearts have increased acetylation and decreased SIRT3 activity, resulting in increased acetylation of several important metabolic enzymes, including LCAD, MCAD, AceCS2, and the ETC [28, 31]. The reduction in SIRT3 activity is likely due to insufficient NAD^+^ bioavailability as a result of dysfunctional energy metabolism, and possibly by direct oxidative modification of the native protein. The impact of abnormal acetylation in the FRDA heart has not been investigated. Because of the important role that acetylation plays in cardiac function, we believe that loss of SIRT3 and resultant mitochondrial protein hyper-acetylation contributes to the heart disease of FRDA.

The purpose of the present study was to document the changes in physiology and function that evolve over time in a model of FRDA heart failure using both non-invasive (ECHO) and invasive left heart catheterization, and match the trajectory of cardiac pathophysiology to the protein acetylation profile along the course of disease in order to determine the link between acetylation of mitochondrial proteins and FRDA heart disease.

## Results

### Cardiac hypertrophy and diastolic dysfunction are early and persistent findings

We used an established conditional mouse model of FRDA heart failure with absence of FXN in the heart (FXN KO) compared to healthy littermates (FXN^*fl/fl*^) [32]. We examined cardiac function in detail, using ECHO and invasive left heart catheterization at postnatal age day 30 ±5, 45 ±4 and 65 ±5. These groups represented pre-, mid- and late heart disease according to previous reports [30].

There were no functional or anatomic differences between hearts of FXN KO and control mice at day 30 (Table 1).

**Table 1:** Heart function as measured by ECHO and invasive left heart catheterization for FXN KO and controls at ages 30, 45 and 65 days.

	30			45		65	
	FXN^*fl/fl*^		FXN KO	FXN^*fl/fl*^	FXN KO	FXN^*fl/fl*^	FXN KO
	n = 7		n = 9	n = 7	n = 6	n = 7	n = 7
*CATH*							
Age (day)	30.7 ±1.8	31.9 ±2.9		46.9 ±0.9	44.8 ±0.4**	63.6 ±3.8	64.6 ±3.4
HR (bpm)	605.3 ±85.3	577.1 ±44.6		671.4 ±95.2	580.9 ±81.4	595 ±77.1	643.3 ±105.2
EF (%)	56.0 ±9.8	49.7 ±7.4		40.9 ±11.8	48.8 ±6.7	50.2 ±6.1	25.2 ±9.5***
+dP/dt (mmHg/s)	1015 ±1848	8760 ±962		11882 ±1928	6875 ±1156***	10809 ±1591	5165 ±1101***
-dP/dt (mmHg/s)	-8114 ±1067	-7802 ±817		-9704 ±799	-6087 ±1378***	-9228 ±891	-4099 ±1104***
MaxPwr	107.5 ±31.2	84.7 ±14.5		143.3 ±32.1	67.5 ±20.7***	121.2 ±31.5	37.3 ±14.1***
Tau (Weiss) (ms)	5.3 ±1.0	5.6 ±0.5		4.5 ±0.6	7.8 ±1.2***	5.0 ±0.5	7.8 ±1.0***
Tau (Mirsky) (ms)	8.7 ±1.5	8.4 ±1.0		8.4 ±0.9	11.0 ± 1.3**	8.6 ± 1.0	10.9 ± 1.5**
ESP (mmHg)	88.6 ±10.2	87.4 ±9.7		103.2 ±5.4	87.7 ±11.9*	99.5 ±7.3	65.2 ±11.0***
EDP (mmHg)	11.7 ±4.5	11.0 ±3.3		10.9 ±3.8	13.0 ±5.7	8.8 ±3.0	10.0 ±5.3
ESV (μl)	22.9 ±7.9	23.1 ±5.1		26.6 ±10.7	24.8 ±5.9	26.8 ±6.9	74.7 ±15.7***
EDV (μl)	50.9 ±6.6	45.7 ±7.3		43.6 ±12.2	48.2 ±9.0	53.4 ±7.8	99.6 ±15.2***
	FXN^*fl/fl*^	FXN KO		FXN^*fl/fl*^	FXN KO	FXN^*fl/fl*^	FXN KO
	n = 10	n = 10		n = 11	n = 10	n = 9	n = 9
*ECHO*							
BW (g)	15.6 ±3.0	13.5 ±3.7		20.4 ±2.0	21.3 ±1.7	23.7 ±2.4	22.4 ±1.2
EF (%)	55.0 ±10.1	54.2 ±7.3		49.9 ±13.7	45.9 ±8.0	47.7 ±8.2	23.9 ±12.5***
FS (%)	28.2 ±6.4	27.6 ±5.0		25.6 ±9.2	22.7 ±4.6	23.7 ±4.9	11.1 ±6.0***
CO (ml/min)	19.7 ±7.2	18.9 ±8.1		17.4 ±5.1	16.8 ±4.6	21.4 ±4.9	13.2 ±5.1*
CI (ml/min/g)	1.2 ±0.3	1.4 ±0.4		0.87 ±0.27	0.80 ±0.24	0.89 ±0.16	0.58 ±0.23*
LVIDd (mm)	3.6 ±0.3	3.6 ±0.4		3.9 ±0.3	3.9 ±0.4	3.91 ±0.14	4.49 ±0.56**
LVPWd (mm)	0.61 ±0.12	0.58 ±0.14		0.64 ±0.08	0.82 ±0.12***	0.62 ±0.13	0.85 ±0.22*
RWT	0.35 ±0.10	0.32 ±0.10		0.33 ±0.05	0.42 ±0.07**	0.32 ±0.07	0.39 ±0.13
LV:body	4.1 ±1.4	4.5 ±1.6		3.9 ±0.7	5.1 ±0.9**	3.47 ±0.70	5.71 ±1.19***
E/A	1.6 ±0.2	1.7 ±0.2		1.5 ±0.2	2.4 ±0.8**	1.4 ±0.2	3.7 ±1.4***
IVRT (ms)	25.1 ±3.0	23.6 ±3.4		23.9 ±2.3	26.8 ±1.2**	21.9 ±2.5	33.6 ±4.2***

Statistical significance between groups based on student’s t-test analysis:

* = *p*<0.05,

** = *p*<0.01,

***p = <0.001.

HR = heart rate; EF = ejection fraction; +dP/dt = ventricle rate of contraction; -dP/dt = ventricle rate of relaxation; maxPwr = maximum power left ventricle; Tau (Weiss) = regression of log(pressure); Tau (Mirsky) = time required for LV pressure to fall to one-half of its value at end systolic pressure; ESP = end systolic pressure; EDP = end diastolic pressure; ESV = end systolic volume; EDV = end diastolic volume; BW = body weight; FS = fractional shortening; CO = cardiac output; CI = cardiac index (CO/body weight); LVIDd = left ventricular internal diameter in diastole; LVPWd = left ventricle posterior wall thickness in diastole; RWT = relative wall thickness (2*LVPWd)/LVIDd); E/A = mitral blood flow velocity ratio of *E*arly-to-*A*trial waves; IVRT = isovolumic relaxation time.

Cardiac hypertrophy is manifest by day 45 in FXN KO animals (Table 1). Compared to controls, day 45 FXN KO mice have thickened left ventricular (LV) posterior walls in diastole (LVPWd) (FXN KO = 0.82 ±0.12 mm vs. FXN^*fl/fl*^ = 0.64 ±0.08 mm; *p*<0.001), increased relative wall thickness (RWT) (FXN KO = 0.42 ±0.07 vs. FXN^*fl/fl*^ = 0.33 ±0.05; *p* = 0.002). FXN KO at day 45 also show findings consistent with both cardiomegaly and hypertrophy with increased ECHO-derived LV:body (FXN KO = 5.1 ±0.9 vs. FXN^*fl/fl*^ = 3.9 ±0.7; *p* = 0.004) and gross heart (mg):body weight (g) ratios (FXN KO: 5.90 ±1.70 vs. FXN^*fl/fl*^: 4.41 ±0.42; *p* = 0.021).

**Fig 1:**
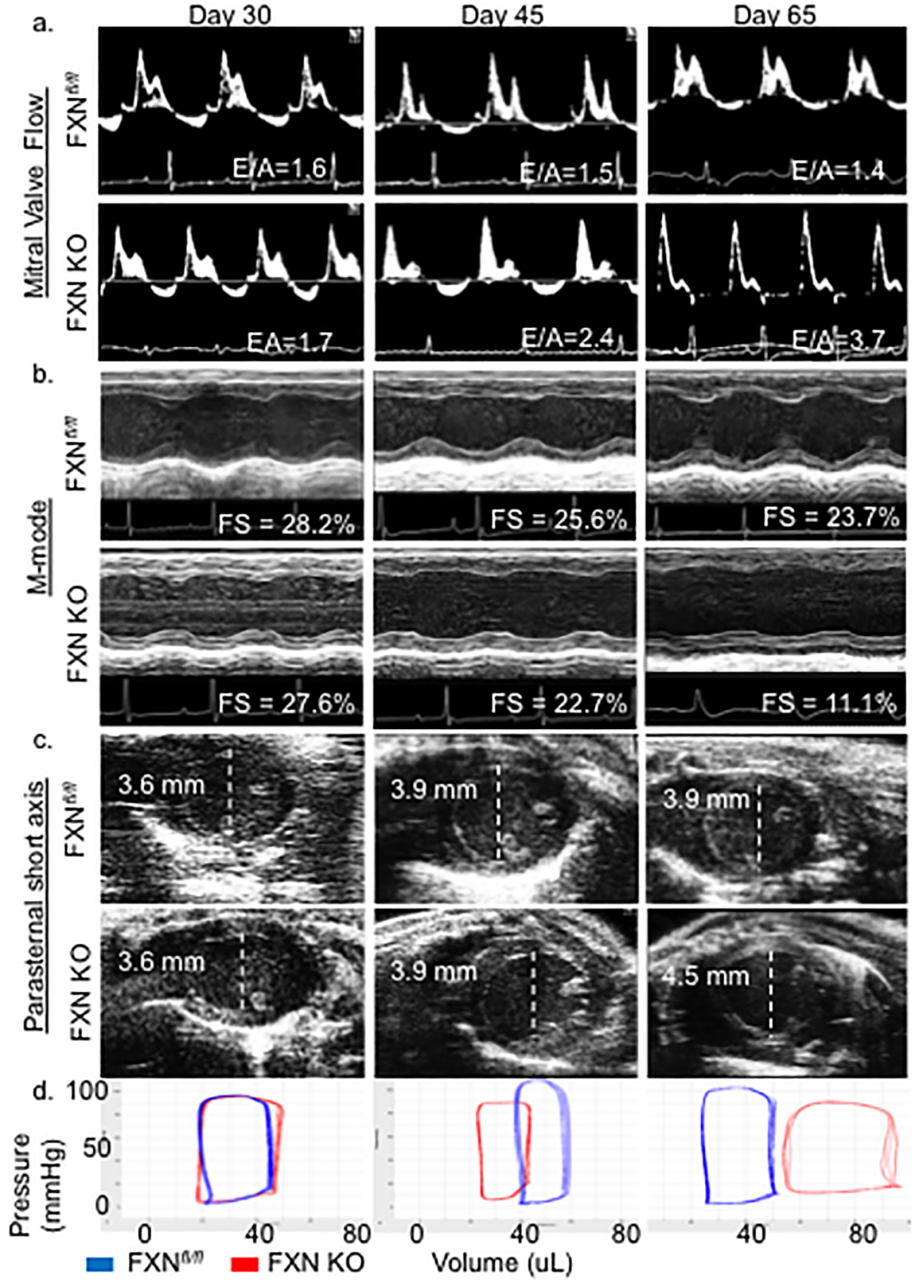
FXN KO mice exhibit diastolic dysfunction followed by dilated cardiomyopathy and heart failure. (a) Representative mitral valve Doppler flow patterns (ratio of the early (E) to late (A) ventricular filling velocity, E/A) demonstrate restrictive cardiomyopathy in FXN KO at days of age 45 and 65. (b) Echocardiographic parasternal short axis M-mode images demonstrate progressive impairment in left ventricular wall movement with decreased fractional shortening (FS, %) in FXN KO. (c) Parasternal short axial images illustrate the transition to dilated cardiomyopathy with increased left ventricular internal diameter in diastole (LVIDd) in FXN KO at day 65. (d) FXN KO pressure volume loops represent increased end-diastolic volume (EDV) at day 65 compared to controls (*p*<0.001) with notable rightward shift of pressure-volume curves. Values indicated in (a), (b) and (c) are averages per group.

Diastolic dysfunction occurs early, is concomitant with presentation of LV hypertrophy, and is persistent in FXN KO hearts. FXN KO mice demonstrate multiple relaxation abnormalities at day 45 (Fig 1, Table 1). Mitral blood flow velocity ratio of *E*arly-to-*A*trial waves (E/A) on Doppler imaging is increased at day 45 (FXN KO = 2.4 ±0.8 vs. FXN^*fl/fl*^ = 1.5 ±0.2; *p* = 0.003) and 65 (FXN KO = 3.7 ±1.4 vs. FXN^*fl/fl*^ = 1.4 ±0.2; *p*<0.001), as well as isovolumic relaxation time (IVRT) at day 45 (FXN KO = 26.8 ±1.2 ms vs. FXN^*fl/fl*^ = 23.9 ±2.3 ms; *p* = 0.008) and day 65 (FXN KO = 33.6 ±4.2 ms vs. FXN^*fl/fl*^ = 21.9 ±2.5 ms; *p*<0.001). Left ventricle relaxation rate (-dP/dt) is decreased in FXN KO compared to controls at days 45 (FXN KO = -6,087 ±1,378 mmHg/sec vs. FXN^*fl/fl*^ = -9,704 ±799 mmHg/sec; *p*<0.001) and 65 (-4,099 ±1,104 mmHg/sec vs. FXN^*fl/fl*^ = -9,228 ±891 mmHg/sec; *p*<0.001). Finally, Tau, the LV relaxation time constant, is increased in FXN KO compared to controls at both day 45 (Tau Weiss: FXN KO = 7.81 ±1.22 vs. FXN^*fl/fl*^ = 4.53 ±0.57 ms, *p*<0.001) and 65 (Tau Weiss: FXN KO = 7.81 ± 0.95 vs. FXN^*fl/fl*^ = 4.99 ±0.50 ms, *p*<0.001), demonstrating prolonged relaxation phase in diastole.

### FXN KO mice transition to dilated cardiomyopathy and heart failure

Contractility abnormalities occur in FXN KO mice as early as postnatal day 45 on invasive measurements, with progressive decline in systolic indices on day 65 measurements (Fig 1, Table 1). At day 45, FXN KO mice have a significant decrease in maximum ventricular power (maxPower) compared to controls (FXN KO = 67.5 ±20.7 mWatt vs. FXN^*fl/fl*^ = 143.3 ±32.1 mWatt; *p*<0.001) as well as rate of contraction (+dP/dt) (FXN KO = 6,875 ±1,155 mmHg/sec vs. FXN^*fl/fl*^ = 11,882 ±1,928 mmHg/sec; *p*<0.001). Again, at day 65, FXN KO mice demonstrate significantly depressed contractility (+dP/dt) (5,165 ±1,100 mmHg/sec) compared to control mice (10,809 ±1,591 mmHg/sec) (*p*<0.001) in addition to decreased maxPower (FXN KO = 37.3 ± 14.1 mWatt vs. FXN^*fl/fl*^ = 121.2 ± 31.5 mWatt; *p*<0.001).

FXN KO mice progress to overt systolic heart failure on ECHO by postnatal day 65. Compared to controls, FXN KO animals have significantly reduced EF (FXN KO = 23.9 ±12.5% vs. FXN^*fl/fl*^ = 47.7 ±8.2%, *p*<0.001) and fractional shortening (FS) (FXN KO = 11.1 ±6.0% vs. FXN^*fl/fl*^ = 23.7 ±4.91%, *p*<0.001). FXN KO mice at day 65 also demonstrate reductions in both cardiac output (CO) (FXN KO = 13.2 ±5.1 ml/min vs. FXN^*fl/fl*^ = 21.4 ±4.9 ml/min, *p* = 0.010) and cardiac index (CI) (FXN KO = 0.58 ±0.23 ml/min/g vs. FXN^*fl/fl*^ = 0.89 ±0.16 ml/min/g, *p* = 0.014) compared to controls.

Consistent with previous reports [30, 32], dilated cardiomyopathy is present in the FXN KO at day 65. There is a significant increase in LV internal diameter in diastole (LVIDd), in FXN KO (4.49 ±0.56 mm) compared to FXN^*fl/fl*^ (3.91 ±0.14 mm)(*p* = 0.006), providing evidence of a dilated LV. Further, the end-cycle blood volumes of the chamber in diastole are markedly increased in FXN KO at 65 days. Left ventricle end-diastolic volume (EDV) data collected with catheter-based direct volume measurements show that day 65 FXN KO mice have an increased EDV that is almost twice that of FXN^*fl/fl*^ (99.7 ±15.2 μl vs. 52.7 ±8.29 μl, respectively; *p* <0.001), causing a dramatic rightward-shift in their pressure-volume curves (Fig 1, Table 1).

FXN KO mice at 65 days of age maintain earlier findings of cardiomegaly on ECHO-derived LV:body weight ratios (5.71 ±1.19) compared to FXN^*fl/fl*^ mice (3.47 ±0.70) (*p*<0.001). FXN KO mice at day 65 also demonstrated close to a 50% increase in gross heart:body weight ratio (8.81 ±1.5) than that measured at day 45 (5.90 ±1.7), and significantly higher than age-matched controls (4.70 ±0.26) (*p*<0.001).

### Acetylation is rapidly and temporally progressive and strongly correlates with a decline in heart function

We previously demonstrated that acetylation in the conditional FXN KO heart is dramatically increased in late stages of heart disease and that the majority of protein acetylation was localized to mitochondria [28]. Here, we examined the level of lysine acetylation in heart tissue at each time point in which we measured cardiac function in order to determine the level of cardiac protein lysine acetylation relative to the evolution of cardiac dysfunction from pre-disease to overt heart failure.

**Fig 2:**
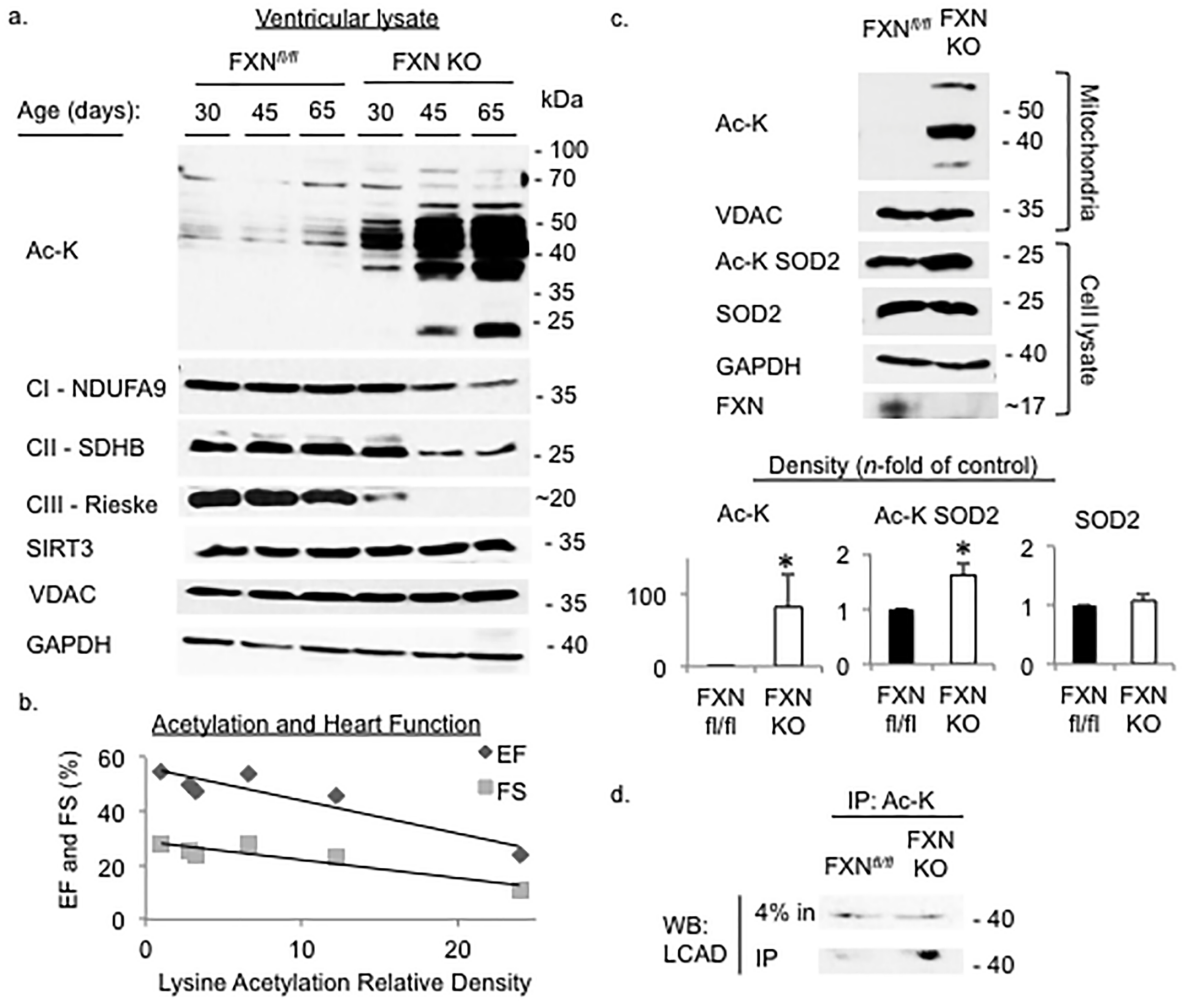
Acetylation is increased and progresses with age in FXN KO, and correlates with worse heart function. Whole cell preparations or mitochondrial isolates from ventricular tissue were probed for proteins of interest as indicated. (a) Acetylation of lysine residues is increased early and progresses with age, and expression of electron transport chain complex subunits decreases in FXN KO hearts. (b) Plot of correlation between acetylation and systolic indices of heart function shows negative correlation between level of acetylation and ventricular function. (c) FXN hearts at day 65 had significantly increased total mitochondrial protein lysine acetylation (*p* = 0.0339), increased acetylation of SOD2 at Lys-68 (AcK-SOD2) (*p* = 0.0016), and (d) increased acetylation of LCAD compared to controls (*p* = 0.0291). EF = ejection fraction, FS = fractional shortening.

We show that acetylation is modestly increased prior to onset of detectable cardiac dysfunction in the FXN KO heart at day 30. This is followed by a dramatically rapid and progressive increase in protein acetylation compared to controls (Fig 2). We measured the expression levels of several essential mitochondrial electron transport proteins, and as expected, based on the established role of FXN in Fe-S cluster enzyme assembly [25, 33] and prior studies [28], the expression of ETC complex subunits CI-NDUFA9, CII-SDBH and CIII-Rieske is reduced in FXN KO and decreases consistently over this time course (Fig 2).

Surprisingly, when the level of acetylation was correlated with measured cardiac function, there was a strong inverse correlation between amount of acetylation, as measured by relative density on western blot imaging, and EF (*r* = -0.923) and FS (*r* = -0.927) (Fig 2).

To demonstrate that mitochondrial deacetylase activity is decreased or absent in the FXN KO hearts, we probed for acetylation of known SIRT3 mitochondrial protein targets, SOD2 and LCAD. SOD2 acts to destroy superoxide radicals and is inhibited when acetylated at lysine 68. SIRT3 reverses acetylation [17, 34]. LCAD is a key enzyme in fatty acid oxidation and reports have demonstrated increased activity in response to deacetylation by SIRT3 [35, 36]. As expected, we detected increased acetylation of both SOD2 and LCAD in the FXN KO compared to controls (Fig 2), confirming decreased activity of SIRT3 deacetylase. These results are consistent with prior studies in our lab that demonstrated hyper-acetylation of SIRT3 targets in cardiac-specific FXN KO hearts, including AceCS2, LCAD, and MCAD [31].

### Abnormal cardiac mitochondria ultrastructure is accompanied by mitochondrial respiratory inhibition

**Fig 3:**
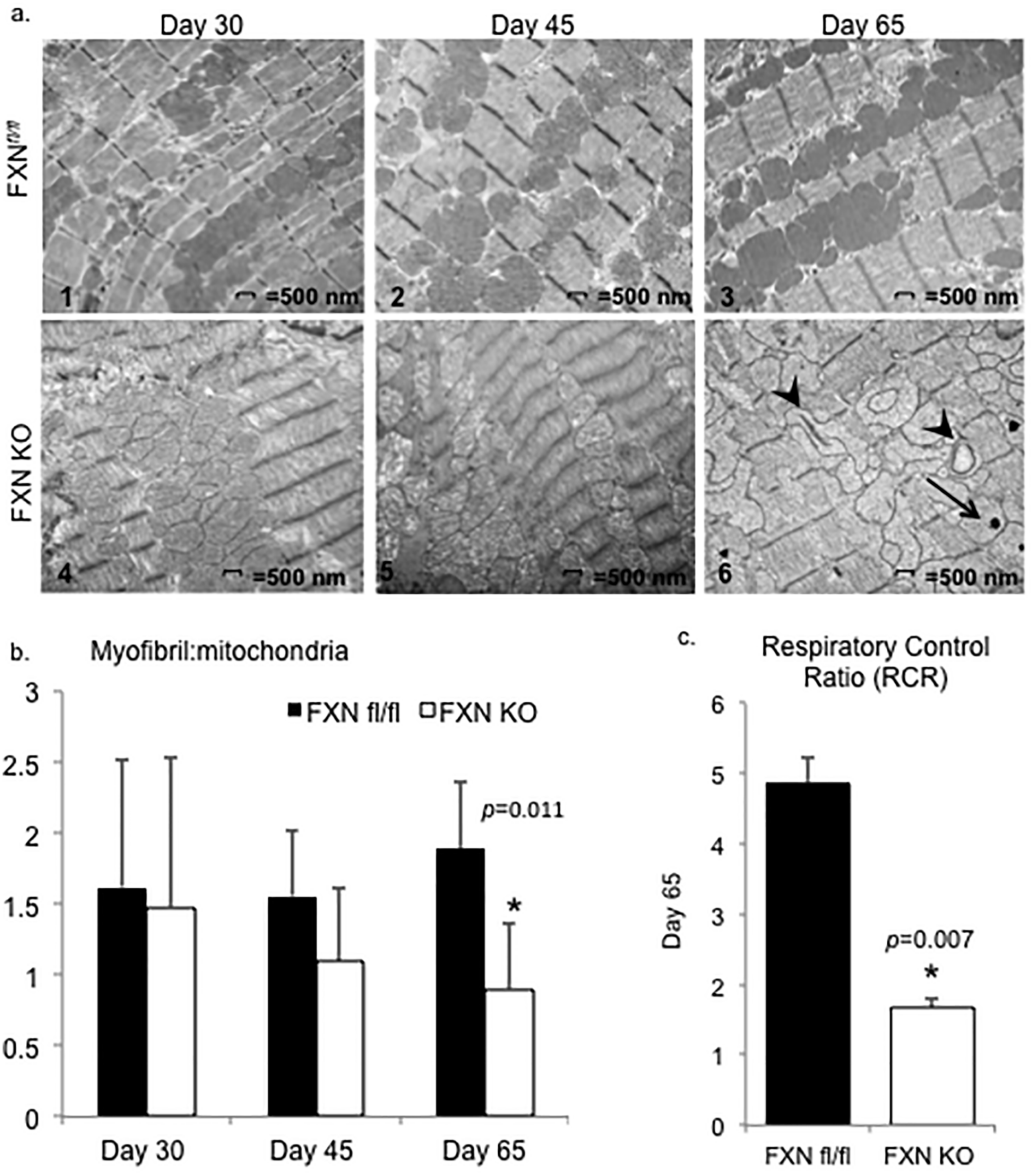
Abnormal cardiac mitochondria ultrastructure is accompanied by respiratory inhibition in FXN KO hearts. (a) Representative images from electron micrographs of cardiomyocytes viewed at 11,000x. Mitochondrial ultrastructure abnormalities were apparent in FXN KO sections and progressed from days 30 to 65. Findings included matrix density loss, mitochondrial-to-sarcomere disarrangement, and accumulation and clumping of mitochondria. FXN KO day 65 mitochondria demonstrate cristae collapse and dissolution, and hyperdense inclusions. Arrowhead = collapsed cristae; arrow = electron-dense intramitochondrial inclusions. (b) Myofibril:mitochondria area ratios were significantly decreased at day 65 in FXN KO compared to controls (n = 3–8 micrographs per strain at each age). (c) Mitochondrial functional assays demonstrated significantly decreased respiratory control ratios in FXN KO compared to controls. There were 2–4 assay runs on pooled mitochondria from FXN KO (n = 8–12 hearts), or pooled mitochondria from controls (n = 4–6 hearts), with 2–3 pooled hearts per run.

Consistent with previous reports [30, 32], we found that pathologic changes in mitochondria become evident on electron microscopy (EM) as early as postnatal day 30 in ventricular tissue of FXN KO, and increase in prevalence over time (Fig 3). Mitochondrial ultrastructure changes in FXN KO hearts include loss and collapse of cristae, disordered mitochondria-to-sarcomere arrangement, and extensive accumulation and stacking of mitochondria, in addition to widespread electron-dense inclusions at day 65.

We quantified these observed morphological changes by measuring the ratio of myofibril-to-mitochondria area from micrographs, and determined the percentage of abnormal mitochondria in each strain [37]. At day 65, the myofibril-to-mitochondria ratio was significantly decreased in FXN KO (0.90 ± 0.46) compared to controls (1.89 ±0.47) (*p* = 0.011), and the average percentage of mitochondrial area containing abnormal mitochondria in FXN KO (78.1±26.7%) was significantly higher than in control micrographs in which no abnormalities were detected (*p* = 0.002) (Fig 3).

We next examined mitochondrial respiratory function at day 65. Respiratory control ratios (RCR) in FXN KO cardiac mitochondria (1.67 ±0.12) were significantly decreased compared to controls (4.86 ±0.37) (*p*<0.001) when measured by Clark Oxygen electrodes using complex I substrates glutamate and malate (Fig 3).

### FXN KO hearts exhibit features of maladaptive ventricular remodeling

**Fig 4:**
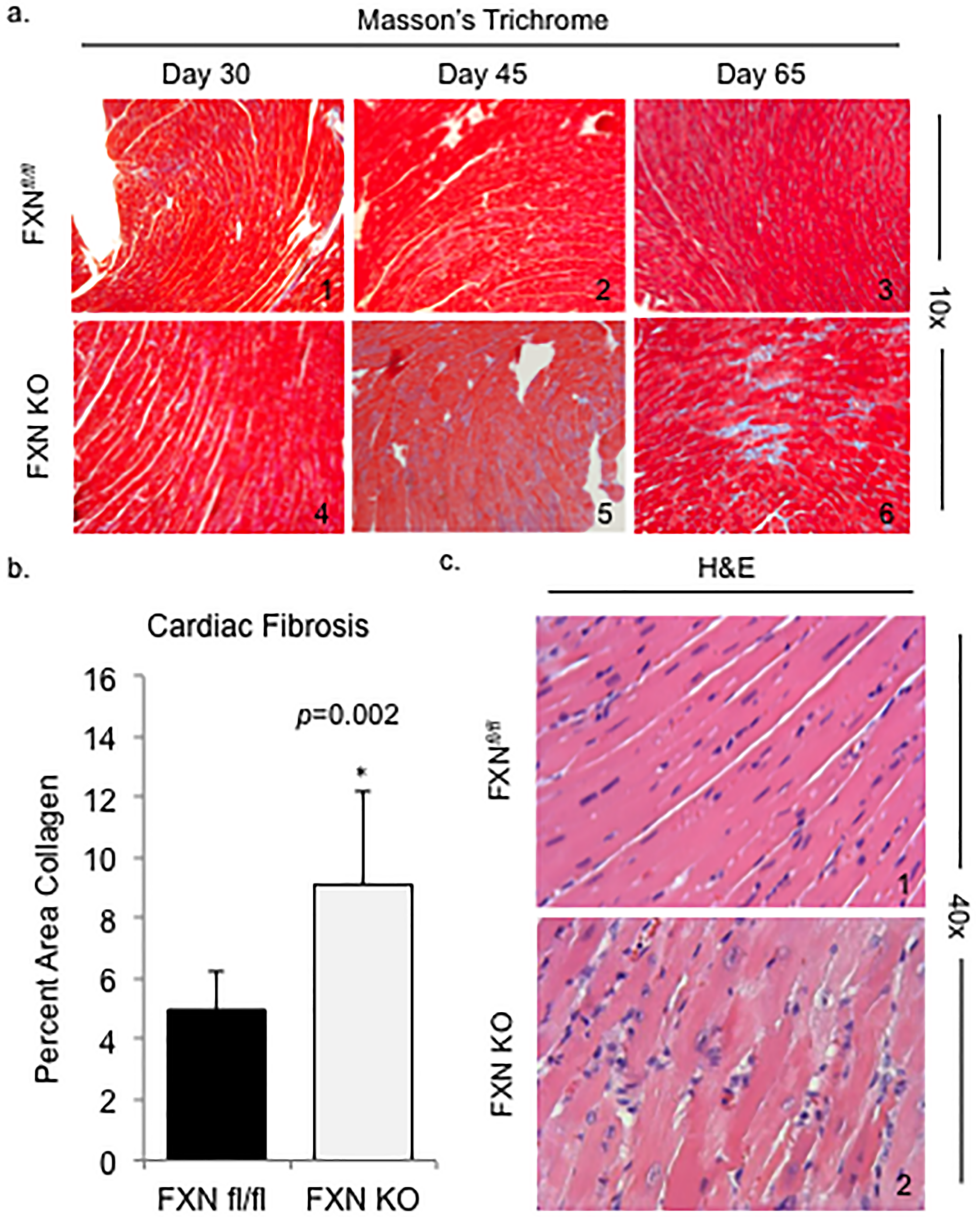
FXN KO hearts exhibit features of maladaptive ventricular remodeling. (a) Cardiac fibrosis is progressive in the FXN KO heart. Ventricular tissue was stained using Masson’s Trichrome to detect blue-staining fibrous tissue. (b) Percent area collagen was significantly increased in FXN KO hearts at postnatal day 65 compared to controls. (c) Evidence of cardiomyocyte degeneration in the FXN KO at day 65 is demonstrated by vacuolation on H&E staining.

Consolidated areas of fibrotic infiltration of the heart are widespread in FXN KO mouse hearts at day 65 (Fig 4), similar to previous reports [32]. By measuring the percent of collagen detected in ventricular tissue on stained micrographs, we demonstrate that FXN KO mice have more than a 1.5 times increase in fibrotic involvement of ventricular tissue compared to controls (FXN KO = 9.1% vs. control 4.9%, *p* = 0.002) (Fig 4). We did not find a significant increase in amount of collagen in the FXN KO hearts compared to controls at days 30 or 45 on histological examination. FXN KO hearts at day 65 also display diffuse cardiomyocyte degeneration, indicated by the presence of vacuolation on light microscopy (Fig 4). Degenerating cardiomyocytes were not detectable at earlier ages (data not shown).

### Loss of FXN in the heart leads to cardiac steatosis and cold intolerance

**Fig 5:**
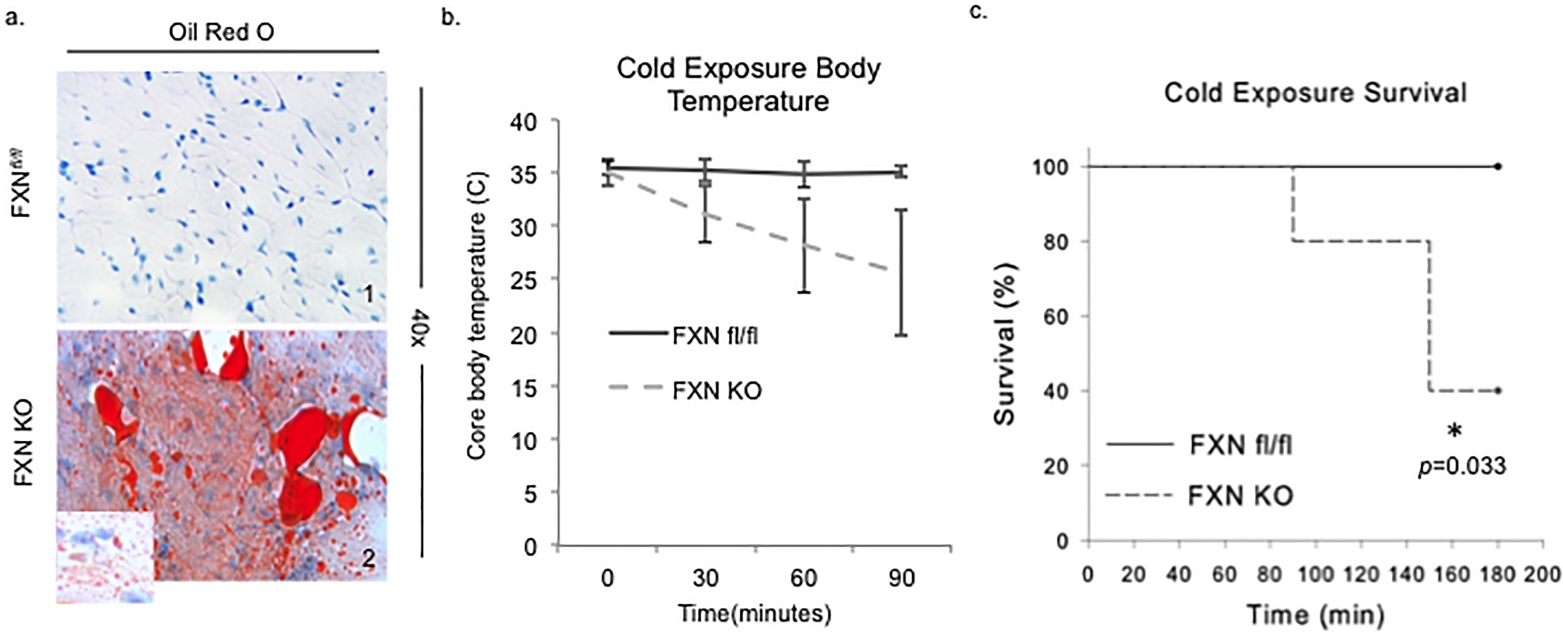
Loss of FXN in the heart leads to cardiac steatosis and cold intolerance. (a) FXN KO hearts demonstrate lipid accumulation in their hearts on oil-red-O staining. (b) Mice were subjected to a 6-hour fast after which they were placed in 4°C room for 3 hours with core body temperature monitoring. (c) FXN KO mice were unable to maintain core body temperature upon cold exposure and suffered significantly increased cold-related mortality rates compared to controls.

Ventricular tissue of FXN KO at day 65 demonstrated a combined pattern of macro- and microsteatosis in cardiomyocytes (Fig 5).

Because mice with loss of MCAD, or VLCAD in the heart quickly become hypothermic when exposed to cold environment and die [38, 39], and, similarly, ablation of SIRT3 leads to cold intolerance [36], we predicted that the conditional FXN KO would be unable to maintain its core body temperature when exposed to a cold stress. Mice were fasted for 6 hours then placed in a cold room at 4°C with core body temperature monitoring every 30 minutes, for a total period of 180 minutes. Indeed, we found that cold exposure lead to a precipitous drop in body temperature and significantly decreased survival rate in the FXN KO animals compared to controls (Fig 5). The majority of the FXN KO animals expired or became moribund with body temperatures <19°C (at which time animals were euthanized for humane reasons) prior to cessation of the three hour time period (Fig 5). Both controls and FRDA mice were observed to be shivering with exposure to cold.

## Discussion

This study is the first to document the progression of lysine-acetylated proteins and demonstrate a correlation between acetylation and cardiac function in the heart of the conditional FXN KO mouse model of FRDA heart disease. We have characterized the initial stages of cardiac dysfunction and evolution to heart failure in this animal model, which mirrors that of the FRDA human heart disease. We conclude that protein lysine acetylation increases in a temporally progressive pattern and that there is a strong negative correlation between level of acetylation in the heart and global heart function: as acetylation increases, ejection fraction and fractional shortening decrease.

We found that abnormal post-translational modification of key metabolic enzymes has occurred prior to detectable impairment in heart function in the FXN KO. Acetylation is increased at day 30 and continues to progress over time. The first notable abnormal traits on cardiac pathophysiology occur at 45 days of age, with thickening of the left ventricular wall and diastolic dysfunction. As acetylation progresses, heart function continues to decline and demonstrates features of decompensated left ventricular dilation, decreased ejection fraction and fractional shortening, consistent with systolic heart failure. These results suggest that targeting acetylation early would provide the best chance to halt or slow progression of FRDA heart disease. Modifying acetylation only after detectable cardiac dysfunction occurs may miss the opportunity to prevent irreversible hemodynamic changes in the FXN KO heart.

Loss of SIRT3 results in maladaptive ventricular remodeling in response to stress [19], abnormalities in lipid metabolism, and reduced tolerance to cold exposure [36]. We found similar outcomes in the FXN KO, which implies that loss of function of SIRT3 in the FXN KO heart and resultant hyper-acetylation of SIRT3 target proteins plays a major role in the pathologic processes of FRDA mitochondrial heart disease. The absence of FXN in the heart, which has an established role in manufacturing of Fe-S cluster subunits needed for enzymes vital to oxidative metabolism, is, in and of itself, a major insult to the cardiomyocyte cellular milieu [40]. The conditional FXN KO model thereby acts as a “stressed heart” model to examine the role of acetylation in heart disease and failure. This is supported by our observation that changes seen in the FXN KO are more robust, with an earlier age at onset, than those seen in models of SIRT3 loss alone. SIRT3 activity is impaired in FRDA hearts likely due to the decreased bioavailability of NAD^+^ and to oxidative damage of SIRT3 [28]. The findings in this study can be applied to other models of heart disease and failure. Many cardiac diseases with abnormal metabolism and energy homeostasis are liable to result in impaired SIRT3 activity in a manner similar to the FXN KO model, leading to abnormal mitochondrial protein acetylation in the heart and impaired cardiac function. Such heart disease candidates include inherited mitochondrial cardiomyopathies, diabetic and metabolic syndrome heart disease, acquired cardiac hypertrophy, age-related and ischemic heart disease, and heart failure [18, 19]. For example, the ischemic heart results in a shift in redox state with accumulation of NADH [41], mirroring the consequential disturbance in energy equivalents in the FRDA heart.

More studies are needed in order to determine a causative role of abnormal acetylation in leading to impairment of hearts with FXN loss. For instance, it would be of great interest to determine whether normalizing the NAD^+^/NADH ratio in the FXN KO heart would stimulate SIRT3 function, recover activity of targeted metabolic enzymes, and improve measured cardiac outcome. Recent work has shown that exogenous treatment with NAD^+^ precursors can increase NAD^+^ levels in mitochondria, reduce protein acetylation, and activate sirtuins [23, 24]. Together with our findings that mitochondrial protein acetylation impairs heart function, this represents an attractive therapeutic potential for pharmacological modulation of protein lysine acetylation to improve cardiac metabolism and physiologic function and prevent unremitting progressive heart disease.

One limitation of this study may be that the MCK promoter used to drive Cre expression in this model is expressed in all sarcomeric tissues, including skeletal muscle. It is not expected that loss of FXN in skeletal muscle would significantly alter cardiac function in this study. Indeed, in patients with FRDA, and in the original report of this mouse model [32], there is not an identified skeletal muscle phenotype. Using the cardiac-specific alpha myosin heavy chain promoter (α-MHC) would address this limitation. However, we determined that the MCK promoter was the logical choice for these experiments both because the α-MHC has also been noted to be cardio-toxic under certain conditions [42], and we wanted to avoid this confounding variable, and because the MCK-Cre transgene has been used extensively in our lab and others’ to generate the FXN KO mouse [28, 32].

In conclusion, we demonstrate a close relationship between mitochondrial protein acetylation, cardiac dysfunction and metabolic disruption in a model of FRDA hypertrophic cardiomyopathy. Our results suggest that abnormal acetylation contributes to the pathophysiology of heart disease in FRDA and may represent a therapeutic target for early intervention.

## Methods

### Mouse breeding and genotyping

This study was approved by the Institutional Animal Care and Use Committee of Indiana University. We used site-specific Cre-lox recombination to carry out gene deletions of interest. We used FXN^*fl/fl*^ mice to create FXN KO mice that were homozygous for heart and skeletal muscle deletion of FXN (MCK-Cre:FXN KO), as previously published [32]. Data was collected from males at postnatal days 30(±5), 45(±4), and/or 65(±5). Controls were age- and sex-matched healthy littermates (FXN^*fl/fl*^).

### Echocardiography (ECHO)

Mice were anesthetized with isoflurane and placed on a warming mat with continuous monitoring. Transthoracic ECHO images were obtained using a VisualSonics^®^ 2100 ultrasound machine for small animal imaging and MS400 transducer (Fujifilm VisualSonics, Inc., Toronto, Canada). Functional parameters of the left ventricle and outflow tracts were measured using standard assessment techniques. Relative wall thickness (RWT) was calculated by (2*LVPWd)/LVIDd).

### Cardiac catheterization

Mice were anesthetized with isoflurane and placed on a warming mat with continuous monitoring. Pressure-volume loops were obtained directly using a 1.2 F microconductance catheter (Scisense, Transonic Systems Inc.), which was inserted into the right carotid artery through a small neck incision and advanced retrograde into the left ventricle. The ADVantage pressure volume system (Scisense, Transonic Systems Inc.) was used to acquire pressure, admittance, phase shift, and amplitude, and real time pressure-volume data was displayed and analyzed offline using specialized software (Labscribe 2, iWorx, Dover, NH) [43].

### Cold stress

This experiment was modeled after similar studies [38, 39]. 65 day old FXN KO (n = 5) and FXN^*fl/fl*^ (n = 6) were selected for cold exposure. Mice were deprived of food for 6 hours prior to onset of study and for the duration of experiment. A baseline rectal temperature was taken prior to placement in 4°C cold room and then every 30 minutes thereafter for a total of 3 hours. A study was terminated for humane reasons if core body temperature reached 19°C or less.

### Histology

Ventricular tissue selected for histology was fixed in 10% formalin and paraffin embedded. Histological analysis included hematoxylin and eosin (H&E) and Masson’s Trichrome to detect collagen as a measure of fibrosis. Collagen quantification was performed using ImageJ (IJ1.46) to measure percent area of tissue positively stained for collagen. Three to five 20x digital micrographs were obtained from each tissue section that underwent quantification studies.

### Electron microscopy (EM)

Ventricular tissue selected for electron microscopy was fixed in 2.5% glutaraldehyde and underwent sectioning and uranyl acetate staining by the Electron Microscopy Center of Indiana University School of Medicine. At least 3 separate sections from each animal strain were selected for quantification analysis. Quantification methods were similar to methods used in our previous work [37]. Abnormal mitochondria were identified as those containing electron-dense inclusions, cristae loss or dissolution, and/or collapsed or condensed cristae. Mitochondria and myofibril area was measured using ImageJ (IJ1.46).

### Isolation of cardiac mitochondria

Mitochondrial isolation followed the method described in our previous work [28]. Freshly harvested hearts were submerged in ice-cold mitochondrial isolation buffer. Heart tissue was weighed, homogenized, and then subjected to differential centrifugation. The mitochondrial pellet was resuspended in the appropriate buffer and used immediately for respiration assay or western blotting, or flash frozen for later analysis.

### Mitochondrial respiration

Mitochondrial isolates designated for use in respiration assays were resuspended in mitochondrial respiration buffer. A total of 2–3 mouse heart mitochondria were combined for each run, with n = 4–6 pooled heart mitochondria assayed for controls and n = 8–12 pooled heart mitochondria assayed for FXN KO. A Clark-oxygen electrode was used for respiration assays. Glutamate and malate were used as a substrate for all assays.

### Antibodies and western blotting

Primary antibodies included anti-acetyl-lysine (Cell Signaling #9441), anti-SIRT3 (Cell Signaling #5490), anti-SOD2 (acetyl K68) (Abcam ab137037), anti-SOD2 (Cell Signaling #13141), anti-VDAC (Cell Signaling #4866), anti-GAPDH (Cell Signaling #5174), anti-FXN (generous gift of Grazia Isaya, Mayo Clinic, Rochester MN), anti-LCAD (Abcam ab196655) and to the electron transport chain complexes: anti-CI-NDUFA9 (Abcam ab14713), anti-CII-SDHB (Abcam ab14714) and anti-CIII-UQCRFS1 (Abcam ab14746). Band intensities were measured using ImageJ (IJ1.46). To determine correlation between acetylation and heart function, relative density of acetylation for n = 3 western blot images was normalized to day 30 controls and averages for each group were used for correlation calculation.

### Statistics

All calculations, analyses and graphs were performed using SigmaPlot (Systat Software, Inc.). Two groups were compared using a two-tailed student’s t test for samples with equal variance. Final data are presented as mean (±SD). Alpha was set at 0.05 and power at 0.800. A *p*-value of <0.05 was considered statistically significant.

## Supporting information

S1 Fig FXN KO mice exhibit left ventricular hypertrophy and diastolic dysfunction and transition to dilated cardiomyopathy and heart failure. FXN KO mice demonstrate cardiac hypertrophy at ages 45 and 65 days on (a) ECHO-derived left ventricle:body mass ratio (LV:body) and (b) left ventricular posterior wall thickness in diastole (LVPWd). Diastolic indices in the FXN KO mice are abnormal; with increases in (c) mitral valve Doppler flow ratio (E/A) and (d) isovolumic relaxation time (IVRT), and (e) decreased rate of left ventricle relaxation (-dP/dt). (f) FXN KO (days 45 and 65) had significantly slower rates of contraction (+dP/dt) and (g) maximum left ventricular power (maxPower) compared to FXN^*fl/fl*^ controls. (h) FXN KO mice demonstrate significantly depressed global contractility function compared to controls for ejection fraction (EF, %) and (i) fractional shortening (FS, %). E/A = ratio of the early (E) to late (A) ventricular filling velocity; -dP/dt = -Δintra-ventricular pressure/Δtime; +dP/dt = +Δintra-ventricular pressure/Δtime. * = *p*<0.05.
(TIF)

S1 File Dataset. (XLSX)
