# Supplementary figures and images for: Progressive mitochondrial protein lysine acetylation and heart failure in a model of Friedreich’s ataxia cardiomyopathy
Source: PLoS One. 2017 May 25;12(5):e0178354. doi: 10.1371/journal.pone.0178354 (PMC5444842; doi:10.1371/journal.pone.0178354)

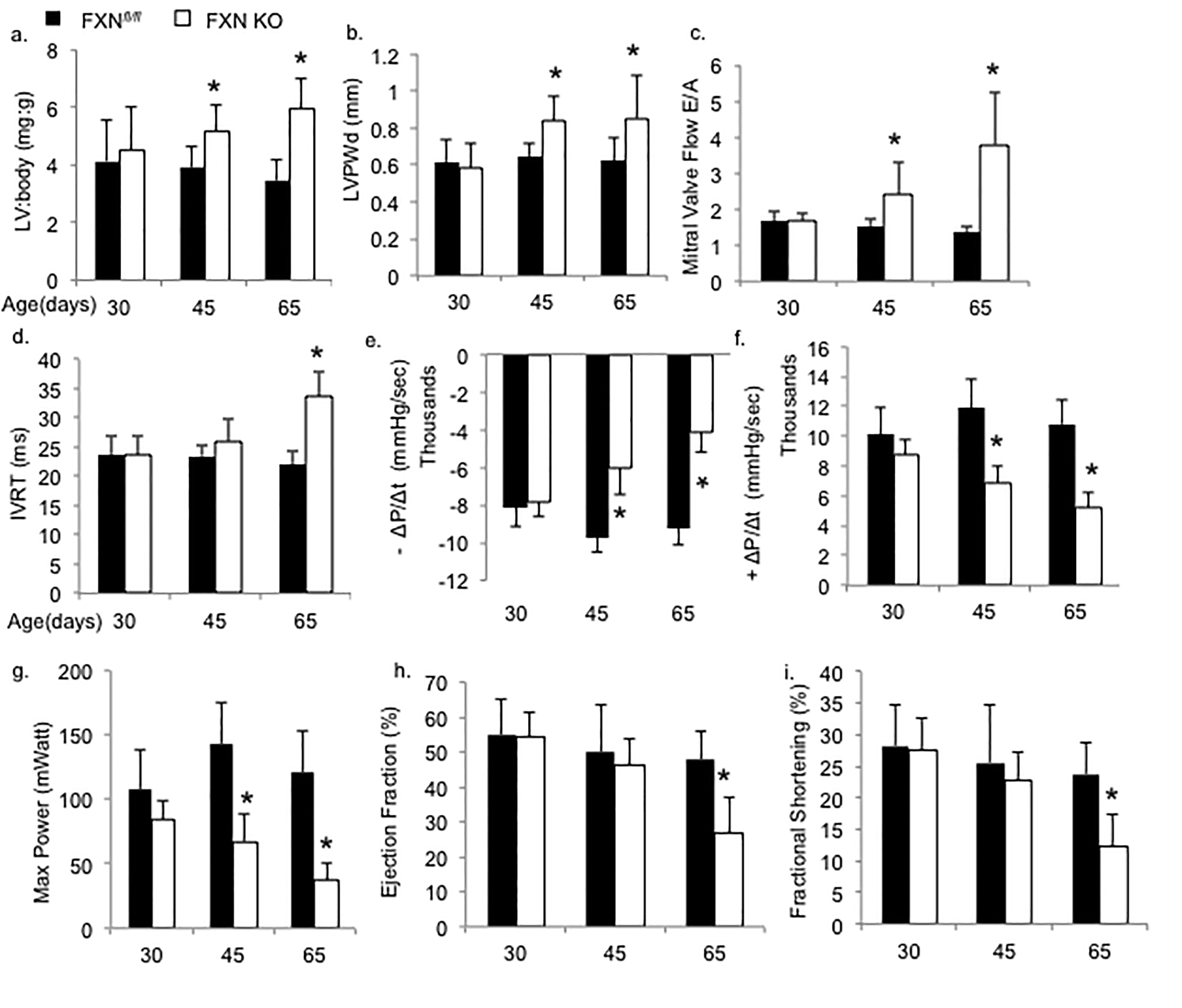

Supplement: S1 Fig — FXN KO mice demonstrate cardiac hypertrophy at ages 45 and 65 days on (a) ECHO-derived left ventricle:body mass ratio (LV:body) and (b) left ventricular posterior wall thickness in diastole (LVPWd). Diastolic indices in the FXN KO mice are abnormal; with increases in (c) mitral valve Doppler flow ratio (E/A) and (d) isovolumic relaxation time (IVRT), and (e) decreased rate of left ventricle relaxation (-dP/dt). (f) FXN KO (days 45 and 65) had significantly slower rates of contraction (+dP/dt) and (g) maximum left ventricular power (maxPower) compared to FXNfl/fl controls. (h) FXN KO mice demonstrate significantly depressed global contractility function compared to controls for ejection fraction (EF, %) and (i) fractional shortening (FS, %). E/A = ratio of the early (E) to late (A) ventricular filling velocity; -dP/dt = -Δintra-ventricular pressure/Δtime; +dP/dt = +Δintra-ventricular pressure/Δtime. * = p<0.05. (TIF) [file pone.0178354.s001.tif]
